# Research on adverse event classification algorithm of da Vinci surgical robot based on Bert-BiLSTM model

**DOI:** 10.3389/fncom.2024.1476164

**Published:** 2024-12-16

**Authors:** Tianchun Li, Wanting Zhu, Wenke Xia, Li Wang, Weiqi Li, Peiming Zhang

**Affiliations:** ^1^School of Health Sciences and Engineering, University of Shanghai for Science and Technology, Shanghai, China; ^2^Henan Center for Drug Evaluation and Inspection, Zhengzhou, Henan, China

**Keywords:** medical device adverse events, Bert-BiLSTM, deep learning, intelligent classification, BERT

## Abstract

This study aims to enhance the classification accuracy of adverse events associated with the da Vinci surgical robot through advanced natural language processing techniques, thereby ensuring medical device safety and protecting patient health. Addressing the issues of incomplete and inconsistent adverse event records, we employed a deep learning model that combines BERT and BiLSTM to predict whether adverse event reports resulted in patient harm. We developed the Bert-BiLSTM-Att_dropout model specifically for text classification tasks with small datasets, optimizing the model’s generalization ability and key information capture through the integration of dropout and attention mechanisms. Our model demonstrated exceptional performance on a dataset comprising 4,568 da Vinci surgical robot adverse event reports collected from 2013 to 2023, achieving an average F1 score of 90.15%, significantly surpassing baseline models such as GRU, LSTM, BiLSTM-Attention, and BERT. This achievement not only validates the model’s effectiveness in text classification within this specific domain but also substantially improves the usability and accuracy of adverse event reporting, contributing to the prevention of medical incidents and reduction of patient harm. Furthermore, our research experimentally confirmed the model’s performance, alleviating the data classification and analysis burden for healthcare professionals. Through comparative analysis, we highlighted the potential of combining BERT and BiLSTM in text classification tasks, particularly for small datasets in the medical field. Our findings advance the development of adverse event monitoring technologies for medical devices and provide critical insights for future research and enhancements.

## 1 Introduction

With the development of society and the progress of medical science, medical devices have gradually become an indispensable part of protecting people’s lives, and their safety has received more and more widespread attention. Although medical devices undergo strict safety assessment and supervision before they are put on the market, there are still certain risks. After they are put on the market, these devices may cause damage to human health. Therefore, continuous quality monitoring and collection of adverse events are necessary. The reporting and analysis of adverse events provide key data for regulatory agencies and help monitor medical devices after they are put on the market. In recent years, due to the continuous improvement of the automation and intelligence of the surgical process, the proportion of the use of da Vinci surgical robots has continued to grow, and the resulting large amount of adverse event texts carry rich data information. In order to obtain valuable information from these adverse events in a timely manner, it is of good practical value to quickly and accurately classify these adverse event texts.

The primary objective of this study is to classify small datasets, specifically focusing on adverse events associated with the da Vinci surgical robot. We selected data from the FDA’s MAUDE database as our main source. The novelty of this research lies in the introduction of a deep learning model that combines BERT and BiLSTM, referred to as Bert-BiLSTM-Att_dropout, specifically tailored to the text classification of medical device adverse events. This innovative approach leverages the powerful contextual information capture capabilities of the BERT model while enhancing the processing of sequential data through the BiLSTM model, complemented by attention mechanisms and dropout strategies to improve the model’s generalization ability and sensitivity to key information.

Compared to existing studies, this research offers unique contributions in several respects. First, it presents a novel classification method specifically designed for the text data related to the da Vinci surgical robot, an area that has not been thoroughly explored in previous research. Second, by incorporating attention mechanisms within the model, this study enhances the precision in identifying and processing key information within the text, which is particularly critical in complex medical text analysis. Lastly, the experimental validation of the model’s efficacy demonstrates outstanding classification performance on small datasets, achieving an average F1 score of 90.15%, a result that stands out in the literature. Moreover, the significance of this study extends beyond improved accuracy and efficiency in classifying adverse events related to medical devices; it alleviates the burdensome data classification workload for healthcare professionals, allowing them to focus more on patient care and surgical procedures. Accurate classification of adverse events also facilitates the timely identification and prevention of serious medical incidents that may pose risks to patients, thereby enhancing the overall safety of surgical procedures and the reliability of medical devices. By advancing the technical capabilities of medical device monitoring, this research makes a substantial contribution to the field of medical safety.

In recent years, the effectiveness of machine algorithms in natural language processing (NLP) has been widely demonstrated. Bacanin et al. (2021) introduced a novel chaotic firefly algorithm that enhances the original firefly algorithm through improved exploration mechanisms and chaotic local search strategies. The paper first validates the theoretical performance of the new algorithm on the CEC benchmark test function suite, subsequently applying it to the dropout regularization problem in deep neural networks (DNNs). The results demonstrate that the Chaotic Firefly Algorithm with Enhanced Exploration (CFAEE) exhibits superior performance in identifying the optimal dropout rate, leading to improved classification accuracy in convolutional neural networks (CNNs).Similarly, Malakar et al. (2020) proposed a hierarchical feature selection model based on genetic algorithms to optimize both local and global features extracted from handwritten word images. This model was experimentally validated on a dataset containing 12,000 samples of handwritten Bengali words. The research not only enhanced the efficiency of feature selection by reducing the feature dimensionality by nearly 28%, but also improved the performance of handwritten word recognition techniques through the optimization of the feature set (González-Carvajal and Garrido-Merchán, 2020). BERT is able to leverage pre-trained knowledge to improve performance. Yu et al. (2019) conducted a series of experiments to improve the performance of the BERT-based text classification model and proposed the BERT4TC model, which achieved significant results on multi-class classification data sets when using appropriate auxiliary sentences. Significant performance improvement, compared with typical feature-based methods and fine-tuning methods, reaching new best performance. By constructing auxiliary sentences and utilizing domain knowledge, the performance of the BERT model in text classification tasks can be effectively improved. Khadhraoui et al. (2022) successfully demonstrated the effectiveness and superiority of the CovBERT model on specific NLP tasks by creating new data sets, preprocessing data, fine-tuning the BERT model, and conducting detailed evaluation and comparative analysis. Mohammadi and Chapon (2020) are exploring the performance of different fine-tuning models based on BERT in text classification tasks. By comparing different fine-tuning strategies and model structures, they concluded that the BERT-Base model has a variety of performance capabilities. superiority in text classification tasks and provides guidance on how to effectively utilize BERT for fine-tuning. Chen et al. (2022) aimed to verify the effectiveness of their proposed long text classification method (LFCN model) based on BERT and CNN in the Chinese news text classification task. The proposed long text classification method based on BERT and CNN was used in It shows high accuracy and effectiveness in Chinese news text classification tasks. Çelıkten and Bulut (2021) used the text classification method based on the BERT model to deal with the classification problem of Turkish medical texts. Cai et al. (2020) conducted experiments to verify the performance of their proposed hybrid BERT model (HBLA) combined with label semantics in multi-label text classification tasks. The experimental results showed that the HBLA model outperformed the major evaluation indicators in terms of major evaluation indicators. most existing methods and achieve new optimal performance. Li et al. (2022) the validation further proves the effectiveness and stability of the model. The software sub-classification method proposed by Bu et al. (2023). Cai et al. (2020) has shown obvious advantages and high accuracy in automatic software label construction, automatic update of classification labels, and fine-grained software classification. Zhonghao et al. (2022) pointed out that the Bert-BiLSTM model improved by 2 percentage points compared with the traditional Bert model method, and could effectively and accurately determine the category of earthquake news, thereby helping earthquake emergency rescue decisions. Ge et al. (2021) proposed the Bert-BiLSTM-ATT model uses the Transformer mechanism in BERT to analyze text. Compared with the traditional LSTM, BiLSTM, BiLSTM-ATT, and Bert-BiLSTM models, the experimental results are better. Xiong et al. (2024) proposed that the Bert-BiLSTM model performed well in identifying consumption intentions. Compared with the single BERT model, the average accuracy, recall rate and Micro-F1 value were increased by 3.67, 4.51 and 3.87 %, indicating that the model is very suitable for consumer intention recognition in short text classification tasks. Mithun and Jha (2023) employed an LSTM model augmented with dropout regularization to selectively hide or deactivate certain neurons, thereby reducing the risk of overfitting. This approach was applied to the classification of a small dataset in lung cancer imaging research. Zivkovic et al. (2022) proposed a novel hybrid firefly algorithm designed for the adjustment and optimization of hyperparameters in the XGBoost classifier, aimed at enhancing the accuracy of network intrusion detection. The paper initially validates the improved firefly algorithm on the CEC2013 benchmark instances and conducts a comparative analysis with other metaheuristic algorithms. Experimental results demonstrate the proposed metaheuristic algorithm’s potential in addressing the challenges of machine learning hyperparameter optimization, thereby improving the classification accuracy and average precision of network intrusion detection systems. Similarly, Jovanovic et al. (2023) explored how to tackle IoT security challenges through the optimization of extreme learning machines (ELMs) using metaheuristic algorithms. They introduced an improved arithmetic optimization algorithm for the hyperparameter optimization and tuning of ELMs, enhancing IoT security. The results indicate that the proposed ELM-HAOA method achieved optimal outcomes in both the best and average scenarios.

In summary, we propose a novel classification optimization algorithm, the Bert-BiLSTM-Att_dropout fusion model, which facilitates the efficient handling of small datasets. To streamline the representation of adverse events and contextual features associated with the da Vinci surgical robot, we utilize the BERT model for training text vectors of adverse events, employing the output of the BERT model as input for the BiLSTM network. Additionally, we incorporate attention mechanisms and dropout strategies to achieve effective classification of adverse events related to the da Vinci surgical robot. This approach presents a new integrated model for the extraction and classification of adverse events associated with the da Vinci surgical robot.

The main contributions of this paper can be summarized as follows:

1. We focus on the first step of medical device adverse event monitoring and use NLP technology to classify text, which improves classification efficiency and saves a lot of manpower and cost.
2. We propose a fusion model of Bert model, BiLSTM model, attention mechanism and Dropout regularization processing for classifying long documents in medical device adverse event monitoring.
3. We have completed experimental verification on real datasets, and our model has achieved state-of-the-art performance.

## 2 da Vinci surgical robot

### 2.1 Features of the da Vinci surgical robot

The specificity and regulatory nature of medical devices are determined by their inherent characteristics. The da Vinci surgical robot is an advanced medical device with a wide range of applications, an extensive history of use, and certain associated risks (Intuitive Surgical, 2020). It is employed across various surgical fields, including but not limited to urology, gynecology, thoracic surgery, and gastrointestinal surgery. Its design flexibility and multifunctionality render it a powerful assistant for surgeons performing complex procedures (Aronson, 2001). The da Vinci surgical robot began its integration into medical practice in 2000, and with ongoing technological advancements, its functionalities and performance have continually improved. Years of clinical practice have yielded extensive experience and data, demonstrating its efficacy and reliability in surgical applications (Garg et al., 2021). Despite the significant achievements of the da Vinci surgical robot in the medical field, certain risks remain in practical applications. Robotic operation necessitates specialized training and skills for surgeons; otherwise, it may lead to operational errors or complications (Patel and Tully, 2020). Additionally, technical failures or equipment malfunctions can adversely impact the surgical process.

### 2.2 da Vinci surgical robot label dataset

The data utilized in this study is sourced from the FDA’s MAUDE database, comprising 4,568 adverse event reports related to the da Vinci surgical robot from 2013 to 2023. This dataset includes five annotated FDA medical device adverse event labels: death, injury, device malfunction, other, and not provided. Following consultations with experts in the field of medical regulation, it was determined to classify the data into two categories: those that did not result in patient harm and those that did. This classification includes 2,484 reports of no harm to patients and 2,084 reports of harm. The classification of each entity and its corresponding rationale are detailed in Table 1.

**TABLE 1:** da Vinci surgical robot FDA label dataset.

Category	Quantity	Description
No harm to patients	2,484	The endoscope’s lens was broken before the da Vinci procedure began. No patient injuries were reported.
Harm to patients	2,084	The surgeon inadvertently severed the atrial artery due to incorrect anatomical identification, and the patient subsequently died.

An example snippet from the dataset. Each drug package insert has a corresponding.txt file containing the raw text. The goal of our system is to automatically identify the cause of each word in the da Vinci surgical robot adverse event label and infer that information to determine its appropriate adverse event category.

## 3 Materials and methods

We employed five methods for the identification and classification of adverse events associated with the da Vinci surgical robot: the GRU model, LSTM model, BiLSTM-Attention model, BERT model, and our own developed Bert-BiLSTM-Att_dropout model. These models were trained on the description sections of the data, inferring candidate categories of no harm to patients and harm to patients from the predictions based on these descriptions. Our deep learning approach utilizes word and character embeddings to represent potential mentions. In this section, we will provide a more detailed description of the models and their ensemble. Our BERT-based method enhances contextualized word embeddings.

### 3.1 Bert model

**FIGURE 1:**
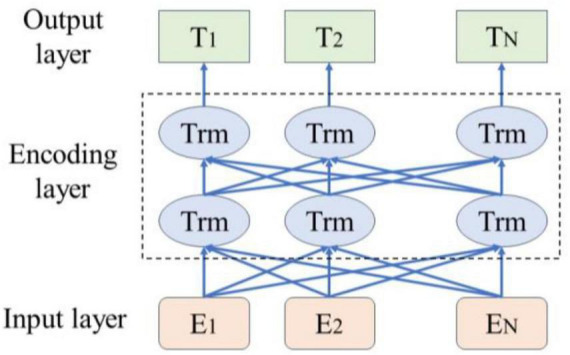
Bert model structure.

As illustrated in Figure 1, the BERT pre-trained language model leverages its bidirectional Transformer architecture to dynamically generate contextual semantic representations of vocabulary. This approach effectively captures sentence features more efficiently than traditional word vector representations, thereby enhancing semantic understanding accuracy (Wang et al., 2019). In 2018, Google introduced BERT, an innovative language model that achieved outstanding results across multiple NLP tasks (Yao et al., 2019). The BERT model employs a Transformer encoder design, characterized by a multi-head self-attention mechanism. This model utilizes a bidirectional encoder to capture contextual information within the text. The input to BERT is a text sequence, formed by combining word embeddings and position embeddings to create input vectors. These input vectors are then stacked through multiple layers of Transformer encoders, resulting in word vectors closely related to their context.

Furthermore, during the pre-training phase, the BERT model performs two key tasks: Masked Language Model (MLM) and Next Sentence Prediction (NSP). The MLM task aims to enhance contextual memory by encoding language. In this process, approximately 85% of the words remain unchanged, 12% are masked using a masking mechanism, 1.5% are replaced with other words, and the remaining 1.5% undergo self-replacement. BERT employs these strategies to construct a bidirectional language model, optimizing language representations by randomly replacing a small number of words through the masking mechanism (Clark and Schmidt, 2013).

**FIGURE 2:**
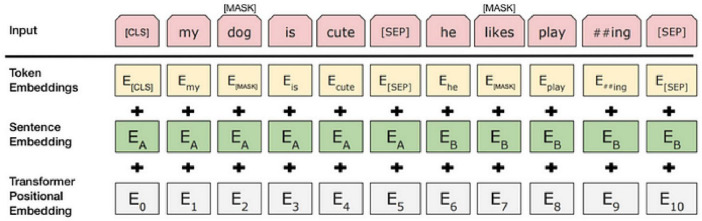
Bert model input flow chart. ##Indicates character separation.

To obtain accurate data, studies typically integrate the contextual background of adverse events associated with the da Vinci surgical robot with the internal text content. By incorporating relevant aspects of the BERT model into the adverse event data for the da Vinci surgical robot, research has demonstrated a significant enhancement in model performance (Wolf et al., 2020). Consequently, as shown in Figure 2, each input token comprises a 768-dimensional token vector, a position vector, and a segment vector. The segment vector has two possible values, indicating whether the segment belongs to sentence A or sentence B. At each token position, these three 768-dimensional vectors are summed to form an input vector, which also has a length of 768; this vector is the actual input to the transformer model (Ni et al., 2020).

**FIGURE 3:**
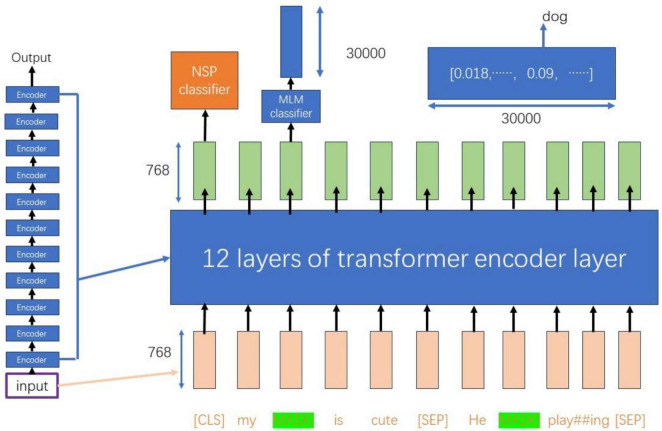
Bert model training flow chart.

As illustrated in Figure 3, the input dimensions of 768 × 11 are processed through 12 layers of Transformer encoder layers, resulting in a new representation of the same dimensions (Hausladen et al., 2020). During this process, the vector of the CLS token is utilized for the Next Sentence Prediction (NSP) task, which serves as a binary classifier to predict whether sentence B is a subsequent sentence to sentence A (Moirangthem and Lee, 2021).

For tokens that have been masked, their 768-dimensional vectors are passed to the Masked Language Model (MLM) classifier, which maps them to a vocabulary of size 30,000. This mapping produces a probability distribution containing 30,000 elements through a softmax layer. This probability distribution can be utilized to infer the possible original forms of the masked words, as well as other potential words at the corresponding positions (Devlin et al., 2019).

### 3.2 Bert-BiLSTM fusion model

**FIGURE 4:**
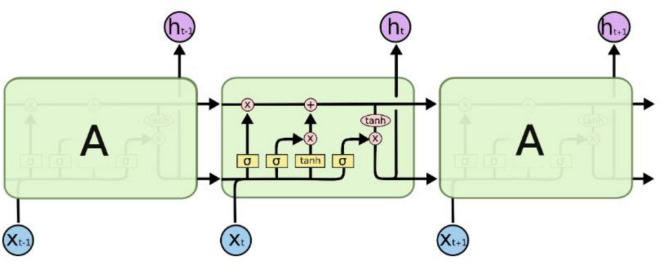
LSTM structure diagram.

Long Short-Term Memory (LSTM) networks, as a specialized form of Recurrent Neural Networks (RNNs), are designed to address the issue of long-term dependencies and are widely adopted due to their excellent capability to handle large samples. Figure 4 illustrates the structure and operational principles of LSTM in detail (Barman and Chowdhury, 2020). LSTM models long-term dependencies through its unique chain-like structure, which, in contrast to traditional RNN models, incorporates four interacting recurrent modules. Each module possesses a specific structural design, distinguishing it from a single neural network layer (Hug and Weil, 2019).

Long Short-Term Memory (LSTM) networks utilize gated structures to precisely regulate the addition and removal of information in the cell state. These gates comprise mechanisms that include sigmoid neural network layers and pointwise multiplication operations, allowing for selective modulation of information flow. LSTM features three key gate structures: the forget gate, the input gate, and the output gate, which serve to protect and control the contents of the cell state.

First, LSTM uses the forget gate to decide which information to discard from the cell state. The output vector*f_t_* of the forget gate is mapped nonlinearly by sigmoid, reads the output *h*_*t*–1_ of the previous time step and the current input *X_t_*, and multiplies it with the cell state *C*_*t*–1_ to decide whether to keep or discard the information in the cell state.


$$ft=\sigma (W_{f}*\left[h_{t-1},x_{t}\right]+b_{f}$$

The next step is to decide which updates to store in the cell state, which has two parts. First, a sigmoid layer, called the input gate layer, decides which values to update.


$$i_{t}=\sigma (w_{i}*\left[h_{t-1},x_{t}\right]+b_{i}$$

Next, the LSTM decides which parts of the cell state to update from the new candidate value *C′_t_* through the input gate. The sigmoid layer of the input gate *i_t_* evaluates *h*_*t*–1_ and *x_t_* while *C′_t_* is generated by the tanh layer.


C′t=tanh(Wc*[ht-1,xt]+bC

Combining these two pieces of information, LSTM updates the cell state *C_t_* by multiplying the old state *C*_*t*–1_ by the forget gate *f_t_* and adding *i_t_* * *C′_t_* to achieve the state update:


Ct=ft*Ct-1+it*C′t

Finally, LSTM decides the output *h_t_* based on the updated cell state *C_t_* The output gate *o_t_* determines which parts of the cell state to output through the sigmoid layer, and converts the cell state to [−1, 1] through the tanh layer and multiplies it by the output of the output gate:


$$o_{t}=\sigma (W_{o}\left[h_{t-1},x_{t}\right]+b_{o}$$

$$h_{t}=o_{t}*\text{tanh}\,\left(C_{t}\right)$$

In this way, LSTM effectively manages and regulates the cell state through the gating mechanism, thus solving the long-term dependency problem in the traditional RNN model.

The BiLSTM neural network structure comprises two independent LSTM input sequences, which are fed into two LSTM networks in both forward and reverse order for feature extraction. Subsequently, the extracted feature vectors are combined to form a single word vector, serving as the final feature representation of that word (Bhavsar and Ganatra, 2016). BiLSTM possesses the capability to train on both past and future information, allowing it to connect output data from the same layer, thereby enabling it to retain historical and prospective context. This approach effectively addresses the issue of traditional LSTM models’ inability to capture contextual information during sequential processing, theoretically enhancing classification accuracy (Jiang et al., 2022).

**FIGURE 5:**
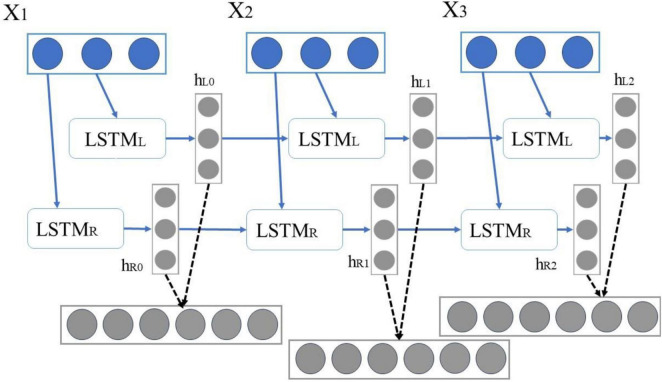
BiLSTM model flow chart.

As shown in Figure 5, in BiLSTM, X1X2X3 is defined as an independent word in a sentence, and its encoding process can be expressed as follows: First, “X1,” “X2,” and “X3” are input to the forward LSTML in sequence, and then three vectors {hl0, hl1, hl2} are obtained. “X1,” “X2,” and “X3” are input to the backward LSTMR in sequence, thereby obtaining three vectors {hr0, hr1, hr2}.

**FIGURE 6:**
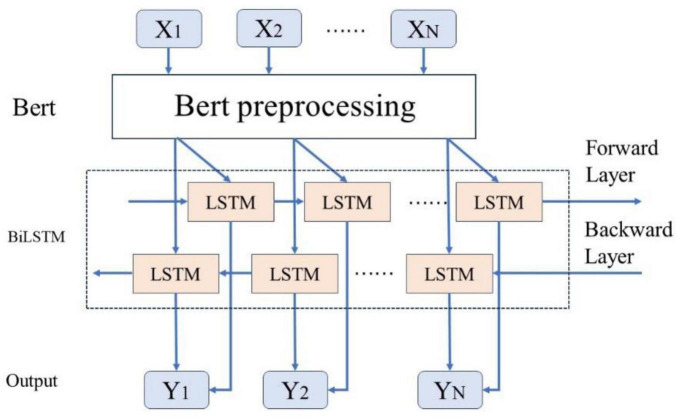
Bert-BiLSTM fusion model diagram.

As illustrated in Figure 6, this study employs a BERT-related language model to train Chinese word vectors, thereby preserving the semantic information of adverse events associated with the da Vinci surgical robot comprehensively. This approach enhances the model’s ability to extract features within context. By utilizing attention mechanisms to encode semantic information, the BiLSTM model can more effectively leverage this information, thereby improving the model’s accuracy in text classification tasks.

### 3.3 Bert-BiLSTM-Att_dropout fusion model

**FIGURE 7:**
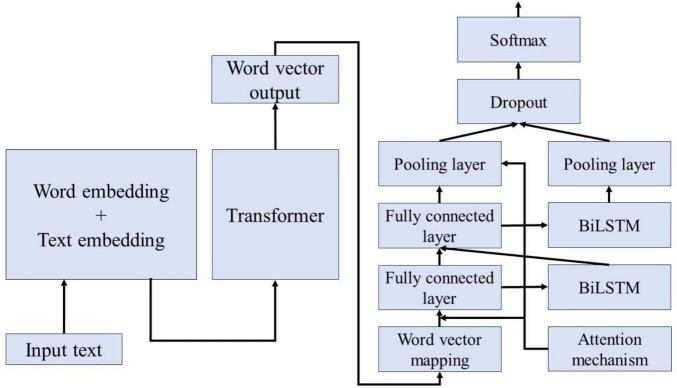
Bert-BiLSTM-Att_dropout model structure diagram.

Figure 7 illustrates the process of extracting and classifying adverse events associated with the da Vinci surgical robot using the BERT-based word vector training and the BiLSTM-Att_dropout model. The preprocessed textual content is input into the BERT model. Following the two pre-training tasks of the BERT model, the adverse event content related to the da Vinci surgical robot is transformed into vector representations. The model’s output consists of the character vectors, text vectors, and position vectors from the adverse event data, integrated to form a comprehensive vector representation of the semantic information. This output is then fed into the LSTM model. The LSTM model combines word vector mapping with a fully connected layer to extract abstract features of the textual information, incorporating an attention mechanism before the fully connected layer to enhance the weight of critical attributes within the text, facilitating feature extraction. This experiment primarily focuses on the binary label text classification task for adverse event categories associated with the da Vinci surgical robot. To address the issue of overfitting in the neural network on the small-scale dataset of adverse events, a Dropout layer is added at the end of the model to extract deep semantic features for classification.

To address the issues of gradient vanishing and contextual significance neglect inherent in the dual LSTM framework, this study incorporates an attention mechanism. By differentiating the importance of various features, the model ignores less significant features while focusing on those that are critical, thereby enhancing classification accuracy. To tackle the challenges faced by BiLSTM, we first compute the similarity and key values of the sequences to establish their weights; subsequently, we apply the Softmax function to normalize these weights. Ultimately, we obtain the final attention values by performing a weighted sum of the weights and the key values.

**FIGURE 8:**
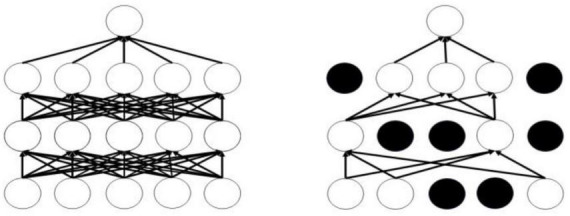
Comparison of before and after using dropout.

The Dropout mechanism aims to address the issue of overfitting in neural networks, thereby enhancing the generalization performance of deep neural networks. Figure 8 illustrates the conditions before and after the application of the Dropout mechanism; the left panel displays the standard neural network structure, while the right panel depicts the layout of the neural network adjusted by the Dropout mechanism. The operational process of the Dropout mechanism is as follows: while ensuring that the input and output neurons remain unchanged, half of the neurons are randomly omitted, and forward propagation is conducted through the adjusted network with the remaining input neurons. Subsequently, the loss results are backpropagated to the respective neurons. After training with a limited number of samples, we utilize stochastic gradient descent to update the parameters of the neurons that were not removed. The neurons that remain are updated, and the previously omitted, unchanged neurons are restored, continuously executing these two steps.

## 4 Experiments

### 4.1 Data preprocessing

The adverse event data related to the da Vinci surgical robot is sourced from the MAUDE database maintained by the US Food and Drug Administration (FDA), comprising a total of 4,568 entries. These data are categorized into two groups: incidents that did not result in harm to patients and those that did. In this study, 20% of the experimental data is designated as validation data, 30% as test data, and 50% as training data, resulting in 913 entries for validation, 1,371 entries for testing, and 2,284 entries for training. The training set constitutes 50% of the total data, ensuring that the model has sufficient data to learn the characteristics and patterns of adverse events. The size of the training set is directly related to the model’s learning capability and performance; a larger training set can aid the model in capturing the complex relationships and patterns present in the data. The validation set accounts for 20% of the data, providing ample information for model selection and hyperparameter tuning. This validation set is used to assess the model’s performance during training, helping to identify the optimal model architecture and parameters while avoiding overfitting to the training set. The test set comprises 30% of the data, offering sufficient information to evaluate the performance of the final model. It is essential that the test set closely resembles unknown data encountered in practical applications to effectively assess the model’s generalization capabilities.

### 4.2 Classification of adverse events of da Vinci surgical robot

**FIGURE 9:**
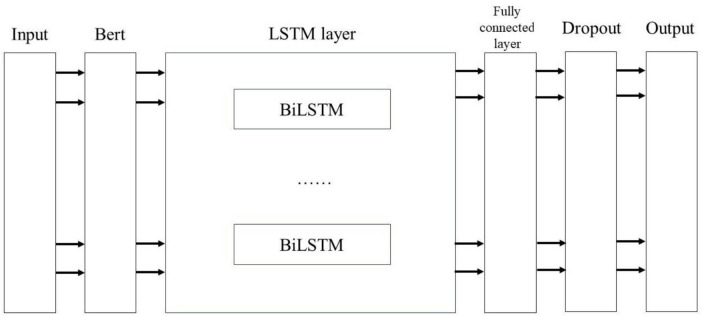
Bert-BiLSTM-Att_dropout model.

In the aforementioned Bert-BiLSTM model, to prevent overfitting and its detrimental impact on the final model’s performance, a dropout regularization operation is incorporated following the Bert-BiLSTM model. This adjustment aims to reduce the risk of overfitting, and its structure is depicted in Figure 9.

**FIGURE 10:**
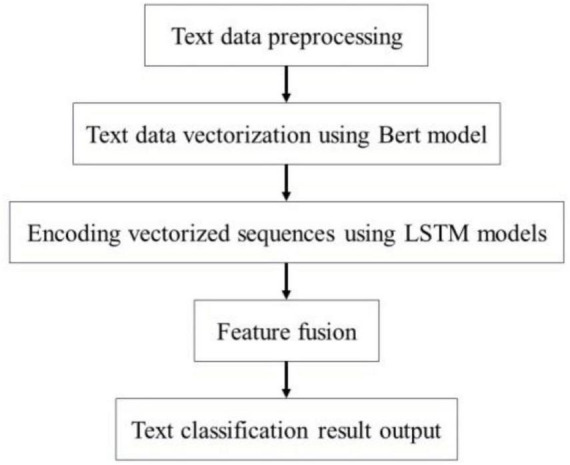
Classification process of adverse events of da Vinci surgical robot.

The Bert-BiLSTM-Att_dropout integrated model refers to the BiLSTM model trained using BERT as the word vector. In the BERT model, we conducted text preprocessing on the adverse event data associated with the da Vinci surgical robot. The processed dataset is then vectorized through the BERT model, which outputs a vector representation that integrates the semantic information of the entire text, subsequently inputting this representation into the BiLSTM model. Next, the BiLSTM model is employed to encode and fuse the features of each sequence, allowing for the extraction of deep semantic features corresponding to each sequence. Through the application of Dropout regularization, we mitigate the overfitting issue of the model, and utilize a Softmax classifier to categorize the obtained deep semantic features. Figure 10 illustrates the detailed steps of the Bert-BiLSTM-Att_dropout model. Overfitting is a common problem for limited datasets, particularly when the model’s complexity is high. When overfitting occurs, the model performs well on the training set but exhibits significantly diminished performance on both the validation and test sets. To mitigate the issue of overfitting, we implemented the following measures:

1. Dropout Regularization: Dropout is an effective regularization technique that reduces the model’s dependence on the training data by randomly omitting a portion of the neurons during the training process. This approach lowers the risk of overfitting. In the Bert-BiLSTM-Att_dropout model, we added a Dropout layer after the BiLSTM layer to further decrease overfitting. 2. Attention Mechanism: The attention mechanism enables the model to focus on key information within the text, thereby enhancing its sensitivity to important features. This mechanism not only improves the model’s performance but also contributes to its generalization capability, as it reduces the likelihood of the model relying on specific noise or details present in the training data. 3. Model Selection: In addition to utilizing the Bert-BiLSTM-Att_dropout model, we conducted comparative analyses with several other models, including LSTM, GRU, BERT, and Bi-LSTM Attention. This comparison aids in understanding the impact of different model architectures on overfitting and in selecting the model best suited to our dataset.

Through these strategies, we effectively addressed the overfitting problem, ensuring that the model maintains robust generalization capabilities even with limited datasets.

## 5 Results

**FIGURE 11:**
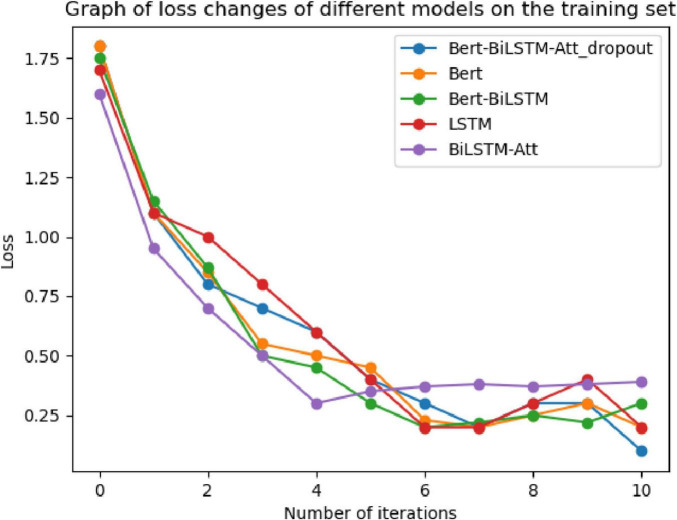
Loss function curve.

**FIGURE 12:**
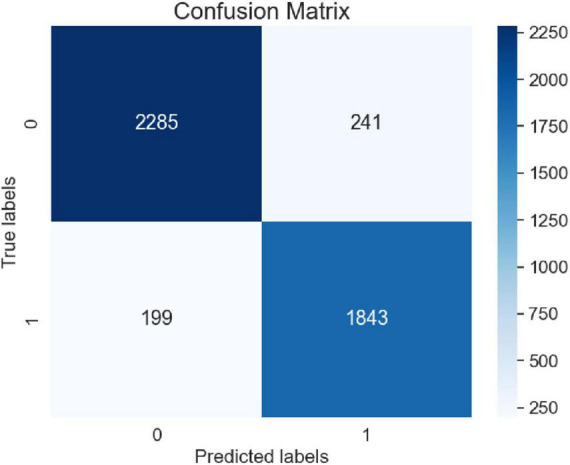
Confusion matrix.

To verify the convergence of the proposed model during the training process, experiments were conducted using the adverse event dataset for the da Vinci surgical robot from the FDA’s MAUDE database. The model underwent ten iterations during training, and the loss values were recorded. These results were compared with four baseline models: LSTM, GRU, BERT, and Bi-LSTM Attention. The comparisons of loss curves and confusion matrices are presented in Figures 11, 12.

We utilized LSTM, GRU, BERT, Bi-LSTM Attention, and our own model, Bert-BiLSTM-Att_dropout, to validate the classification effectiveness of the binary classification task on the da Vinci surgical robot adverse event dataset. This evaluation allowed us to report the accuracy, recall, and F1-score for each classification category. Precision is defined as the ratio of correctly predicted mentions to the total number of predicted mentions for a specific entity; recall is the ratio of correctly predicted mentions to the actual mentions; and the F1-score is the harmonic mean of precision and recall. We also reported average values, calculating metrics independently for each class and then averaging them across all classes. Given the class imbalance in our binary classification task, micro averaging is preferable. The experiments compared five models, with performance metrics detailed in Table 1; under the same conditions, a higher F1 score indicates better model performance. The calculation formulas for these metrics are defined as follows:

Precision: The ratio of positive samples predicted to be true (TP) to all samples predicted to be true:


$$\mathrm{precision}=\frac{\text{TP}}{\mathrm{TP}+\mathrm{FP}}$$

Recall: the ratio of predicted true positive examples (TP) to all samples that are actually positive examples:


$$\mathrm{recall}=\frac{\text{TP}}{\mathrm{TP}+\mathrm{FN}}$$

The calculation formula of F1 parameter is as follows:


$$\mathrm{F1}=\frac{2\,T\,P}{2\,T\,P+\mathrm{FP}+\mathrm{FN}}$$

In this study, the dataset is divided into two categories, each with independent precision, recall and F1 value. The evaluation indicators used in this paper include average precision (*P*-value), average recall (*R*-value) and average F1 value. The calculation formulas of these indicators have been defined in the article, and the corresponding calculation results have been shown in Table 2.

**TABLE 2:** Performance indicators of the models.

		No harm to the patient	Harm to the patient
Precision	LSTM	75.14	77.23
	GRU	77.86	78.59
	Bert	78.03	80.09
	BiLSTM-Attention	84.31	87.11
	Our	91.71	88.31
Recall	LSTM	74.01	77.29
	GRU	74.34	78.56
	Bert	75.13	80.43
	BiLSTM-Attention	88.75	83.07
	Our	90.59	91.07
F1-score	LSTM	73.12	79.16
	GRU	73.89	80.43
	Bert	74.77	82.45
	BiLSTM-Attention	81.19	89.55
	Our	91.46	89.84

The calculation formula for the average accuracy is as follows:


$$P=\left(\frac{1}{n}\right)\,\sum_{i=1}^{n}p\,r\,e\,c\,i\,s\,o\,n$$

The calculation formula for the average recall is as follows:


$$R=\left(\frac{1}{n}\right)\,\sum_{i=1}^{n}r\,e\,c\,a\,l\,l$$

The average F1 value is calculated as follows:


$$A\,v\,e\,r\,a\,g\,e\,F\,1=\left(\frac{1}{n}\right)\,\sum_{i=1}^{n}\frac{2*p\,r\,e\,c\,i\,s\,i\,o\,n*r\,e\,c\,a\,l\,l}{p\,r\,e\,c\,i\,s\,i\,o\,n+r\,e\,c\,a\,l\,l}$$

## 6 Discussions

We compared the classification performance of our model with four benchmark models: LSTM, GRU, BERT, and Bi-LSTM Attention on the da Vinci surgical robot adverse event dataset. In this study, we employed the BERT model to construct word vector representations for the text, serving as the input data for effective classification analysis of adverse events related to the da Vinci surgical robot. Our comparison of LSTM, GRU, BERT, and Bi-LSTM Attention models revealed that the word vectors generated by the BERT model had a significantly positive impact on the classification task. The Transformer layers of the BERT model, with their bidirectional encoder structure, greatly enhanced the memory capacity for text context, thereby optimizing classification performance. Furthermore, we improved the model by integrating attention and dropout mechanisms into the architecture. By calculating time series vectors and applying weighted sums with weighted attention techniques as feature vectors, our model effectively addressed the gradient vanishing problem and the neglect of contextual information that Bi-LSTM models may encounter when processing long sequences. Additionally, the introduced dropout mechanism helped mitigate overfitting, enhancing the model’s generalization capability. Ultimately, the model demonstrated precise categorization corresponding to various selection criteria, underscoring its significant value in practical applications.

Table 2 presents the Precision, Recall, and F1-score metrics. From the Precision section, it is evident that in the category of “no harm to patients,” the Precision of the LSTM and GRU models is slightly lower than that of the BERT model, while the Precision of the Bi-LSTM Attention model and our proposed model is comparatively higher. This indicates that our model is more inclined to accurately predict positive cases when forecasting samples that do not cause harm to patients. In the category of “harm to patients,” the Precision values among the three models are quite similar, with our model showing a slight advantage over the other four models. This suggests that our model demonstrates better accuracy when predicting samples that cause harm to patients. In the Recall section, it can be observed that the Bi-LSTM Attention model achieves the highest Recall in the “no harm to patients” category, while the Recall of the BERT model is marginally lower than that of the other three models. This implies that the Bi-LSTM Attention model is more effective in capturing positive cases in this category. Conversely, our model exhibits the highest Recall in the “harm to patients” category, whereas the LSTM model records the lowest Recall, indicating that our model performs better in identifying samples that cause harm to patients. The F1-score section reveals that the F1-score, which integrates both Precision and Recall performance, serves as a comprehensive evaluation metric. Our model attains the highest F1-score in the “no harm to the patients” category, demonstrating a favorable balance between Precision and Recall. Similarly, in the “harm to the patients” category, our model also achieves the highest F1-score, indicating its superior overall performance in this category. Overall, our model exhibits the best performance in both “no harm to patients” and “harm to patients” categories. This illustrates that across different categories and performance metrics, our model possesses significant advantages and broad applicability, necessitating the selection of an appropriate model based on specific tasks and requirements.

From Table 3, the Precision, Recall, and Average F1 scores indicate that our model outperforms the other models across all metrics. The experimental results demonstrate that the average F1 score on the test set for the single LSTM model is 76.14%, while the proposed model based on GRU achieves an average F1 of 77.16%. The BERT model yields an average F1 of 78.61%, and the Bi-LSTM Attention model reaches an average F1 of 85.37%. In contrast, our model achieves an impressive average F1 of 90.15%. When utilizing word vector models to obtain sentence vectors, the training outcomes for the Bi-LSTM Attention model and our model differ significantly, with our model showing a marked improvement in performance. The Precision section reveals that our model has the highest Precision at 90.01, clearly surpassing other models. This indicates that our model has the highest accuracy in predicting positive cases while maintaining the lowest false positive rate. In the Recall section, our model also demonstrates the highest Recall at 90.83, significantly exceeding the other models. This highlights our model’s superior performance in identifying positive cases, coupled with the lowest false negative rate. Regarding the Average F1 scores, our model achieves the highest Average F1 of 90.15, effectively integrating the performance of Precision and Recall, and thus serving as a comprehensive evaluation metric. This demonstrates that our model strikes an excellent balance between Precision and Recall. Overall, the experimental results confirm that our model outperforms the other models, particularly after the incorporation of the dropout layer, which has led to a significant enhancement in performance. This indicates that our model possesses substantial advantages and broad applicability across different categories and performance metrics.

**TABLE 3:** Comparison of evaluation indicators of models.

Model	*P*-value	*R*-value	Average F1
LSTM	76.19	75.65	76.14
GRU	78.23	76.45	77.16
Bert	79.06	77.78	78.61
BiLSTM-Attention	85.71	85.91	85.37
Our	90.01	90.83	90.15

## 7 Conclusion

The safety of medical devices is a critical factor in ensuring patient health, necessitating ongoing monitoring and evaluation even after these devices are brought to market. However, adverse events related to medical devices remain inevitable. To address the challenges posed by the limited sample size of adverse event data, low utilization of classification information, and difficulties in information extraction, we propose a short text classification model based on the BERT model, specifically the Bert-BiLSTM-Att_dropout integration. Initially, we preprocess the adverse event data related to the da Vinci surgical robot, categorizing it into two groups: events that did not harm patients and those that did. We allocate 30% of the experimental data for testing and 70% for training, followed by sorting and cleaning the annotated data. This study evaluates and compares the performance of the models. For the BiLSTM-Attention model, we utilize BERT word vectors to obtain the text representations of the da Vinci surgical robot’s adverse events, which serve as inputs for the BiLSTM model, ultimately yielding classification results. In the case of the Bert-BiLSTM-Att_dropout model, we describe the training process within the BiLSTM-Att_dropout text classification framework. The experimental results indicate that the Bert-BiLSTM-Att_dropout model achieves the highest average F1 score of 90.15% in classification performance. Furthermore, a comparison with four other models reveals that the classification effectiveness of the Bert-BiLSTM-Att_dropout model is significantly enhanced.

Thus, the proposed Bert-BiLSTM-Att_dropout integrated model demonstrates significant application value in the extraction and classification of adverse events related to the da Vinci surgical robot. However, this study presents certain limitations both theoretically and practically. From a theoretical perspective, while the Bert-BiLSTM-Att_dropout model exhibits impressive performance on small datasets, its effectiveness in handling larger-scale datasets remains inadequately validated. Additionally, the model’s generalizability across diverse domains and languages requires further investigation. From a practical standpoint, the model’s computational complexity is relatively high, necessitating substantial computational resources, which may restrict its application in resource-constrained environments. Furthermore, the model’s heavy reliance on data quality and annotation can pose challenges in real-world scenarios. To address these limitations, future work could involve training and testing the model on larger datasets to assess its performance and generalizability across various scales. Additionally, exploring the model’s applicability to different domains and languages through cross-domain and multilingual experiments could enhance its versatility. Furthermore, research should focus on optimizing the model’s architecture and training process to reduce computational complexity, thereby facilitating deployment in resource-limited settings. Finally, enhancing the model’s robustness against fluctuations in data quality and its performance under conditions of inaccurate annotations should also be prioritized.

## Data Availability

The original contributions presented in this study are included in this article/supplementary material, further inquiries can be directed to the corresponding author.
